# Author reply to letter to the editor: From fragmentation to frameworks: Standardizing AI in gastrointestinal endoscopy

**DOI:** 10.1055/a-2695-2884

**Published:** 2025-09-24

**Authors:** Kristoffer Mazanti Cold, Amaan Ali, Lars Konge, Flemming Bjerrum, Laurence Lovat, Omer Ahmad

**Affiliations:** 1 615551 Rigshospitalet, CAMES, Copenhagen, Denmark; 2 4919 Wellcome/EPSRC Centre for Interventional & Surgical Sciences (WEISS), University College London, London, United Kingdom of Great Britain and Northern Ireland; 3 Centre for Clinical Education, University of Copenhagen and the Capital Region of Denmark, Copenhagen, Denmark; 4 4919 Hawkes Institute, UCL, London, United Kingdom of Great Britain and Northern Ireland; 5 4919 Department of surgery and interventional sciences, UCL, London, United Kingdom of Great Britain and Northern Ireland



We appreciate the Letter to the Editor by Deding et al. 1 : “Urgency for standardized protocols to improve clinical implementation of artificial intelligence (AI) in endoscopic diagnostics”, emphasizing the need for development of AI to follow protocols rather than being fragmented, as summarized in the systematic review 2 the Letter to the Editor addressed 1 . Their emphasis on AI in capsule endoscopy (CE) is timely, especially given the European Union’s support of initiatives such as I-Supported Image Analysis in Large Bowel Camera Capsule Endoscopy (AICE) and in general toward improvements in diagnosing and treating colorectal cancer. CE interpretation remains a labor-intensive task with high interobserver variability. An Endoscopy International Open study suggested that learning small bowel CE may be more difficult and labor-intensive than previously assumed, because none of 22 gastroenterologists reached a learning plateau with sufficient competencies after reviewing 20 small bowel CE, with an accumulated specificity for diagnosis of just 63% and sensitivity of just 65% 3 .

We acknowledge and agree with their concerns regarding the limitations of human-centric reference standards such as the Boston Bowel Preparation Scale (BBPS) to train AI, which is widely used yet inconsistently correlated with clinically relevant outcomes such as adenoma detection rate (ADR), polyp detection rate (PDR), and adenoma miss rate (AMR) 2 . Importantly, one of the eight studies included in our review used a fecal-to-mucosa pixel ratio and was validated against > 1,400 colonoscopies and an external dataset, correlating with PDR rather than just BBPS 4 . In addition, it was the only AI to be open-source, allowing for external validation, as an important part of protocol for validating AI, highlighted by Deding et al. 1 .

In parallel with AICE, through the intelligent robotic endoscopy (IRE) initiative ( https://ire4health.eu/ ), we have published a freely available dataset of over 1,400 clinical colonoscopies and 100 simulated colonoscopies with full colonoscope positional tracking throughout the procedure 5 . This dataset facilitates development of AI systems that incorporate spatial-temporal tracking, particularly relevant for development of new modalities such as robotic endoscopy, through IRE.

In conclusion, we concur with Deding et al. 1 that future AI models should be explainable and validated against hard clinical outcome measures such as ADR, PDR, or AMR, aligning with the recent European Society of Gastrointestinal Endoscopy position statement on the expected value of AI in endoscopy 6 . To that end, reporting guidelines such as Quality assessment of AI preclinical studies in diagnostic endoscopy (QUAIDE) 7 represent a major step forward as a protocol for standardization. We support widespread adoption of such frameworks to ensure standardization, reproducibility, and meaningful clinical implementation of AI in both conventional colonoscopy and CE along with making these AI algorithms and datasets open-source for external validation and training.

**Publication note**

Letters to the editor do not necessarily represent the opinion of the editor or publisher. The editor and publisher reserve the right to not publish letters to the editor, or to publish them abbreviated or in extracts.
